# The usage of over-the-counter products by private insured patients in Germany – a claims data analysis with focus on complementary medicine

**DOI:** 10.1186/s12913-020-05501-1

**Published:** 2020-07-13

**Authors:** Katja Goetz, Matthias Kalder, Ute-Susann Albert, Christian O. Jacke

**Affiliations:** 1 Institute of Family Medicine, University Hospital Schleswig-Holstein, Campus Luebeck, Ratzeburger Allee 16, Building 50, 23538 Lübeck, Germany; 2 Department of Gynecology and Obstetrics, Philipps University of Marburg, Baldingerstr., 35043 Marburg, Germany; 3 Department for Obstetrics and Gynecology, University Hospital Würzburg, Josef-Schneider-Straße 4, 97080 Würzburg, Germany; 4 Scientific Institute of Private Health Insurance (WIP), Gustav-Heinemann-Ufer 74c, 50968 Köln, Germany

**Keywords:** Anthroposophy, Homeopathy, Private health insurance, Pharmacoeconomy, Over-the-counter drugs, Prescriptions

## Abstract

**Background:**

An important contribution to well-being of human beings can be observed by the use of self-medication products that is reflected in the constantly growing volume of over-the-counter (OTC) drugs. The aim of the current study was to extend the measurement concept for OTCs by exploring the relevance of the peripheral assortment provided by the widely accepted framework of the Anatomical Therapeutical and Chemical (ATC) classification of the WHO.

**Methods:**

The focus was on the prescriptions and drug-related receipts submitted by privately insured persons to 18 private health insurers (PHIs) in Germany from the year 2016. The age- and gender-specific average claims amount per risks of outpatient drug expenditure were used as weights to scale up the relative distributions of the item amounts. The ATC-classification defines the commodity groups and discriminates between the main and the peripheral assortment. A descriptive analysis assessed the OTC frequencies and sum scores of the product groups within the main and peripheral assortment whereby the study group explored and assessed the relevance of each category independently according to the OTCs and integrative medicines.

**Results:**

The analysis included 22.1 Mio. packages from the main assortment and examined 10.1 Mio. packages from the peripheral assortment. The latter was examined thoroughly and the commodity groups “Pharmaceutical food products”, “Medicinal products for special therapy options” and particular “Hygiene and body care products” meet the defined requirements for OTCs relevant for integrative medicines. A high proportion of OTC products from the peripheral assortment was associated with the categories “medicinal products for special therapy options”. Homeopathy and anthroposophy present two special therapy options, which are relevant for the extended OTC measurement.

**Conclusions:**

The analysis of OTC drugs is feasible when the main and the peripheral assortment is available and enable to integrate about 18% of all OTCs, which are neglected by the common ATC-based approach. The presented extended approach may help to identify potential users of OTCs or people in need of OTC use. In case of the highly disputed homeopathy and anthroposophy products, more research among interactions with prescriptions drugs (Rx), nutrition’s and other potentially harmful exposures is recommended.

## Background

An important contribution to health and well-being of human beings can be observed by the use of self-medication products [1]. Self-medication is a widespread phenomenon and gains increasing importance in terms of taking responsibility for their own health. An expression of this development is reflected in the constantly growing volume of over-the-counter (OTC) drugs [2]. People show an increased willingness to pay, even if this means higher out-of-pocket expenses for drugs of partly dubious medical evidence. In general, self-medication can facilitate access to medicines and reduce health care costs for payers [3]. The Association of the European Self-Medication Industry (AESGP) stated that self-care saves the European health care system more than 16 billion Euros [4]. Beside the economic benefit, it can be assumed that preventive and curative aspects are the main causes for the increased utilization [5].

### Current academic efforts in Germany

The importance of complementary respectively integrative medicine is also recognized by the required academic research facilities, which have been recently funded. A further step to provide the requested evidence presents the registration of a S3-guideline “Complementary medicine in treatment of oncological patients” by the Association of the Scientific Medical Societies (AWMF) in Germany [6]. The dissemination of the updated guideline is expected at the end of 2020. Furthermore, different international guidelines exist already. For example the Society for Integrative Oncology published a clinical practice guideline on use of integrative therapies during and after breast cancer treatment [7, 8]. The American College of Chest Physicians produced the guideline of complementary therapies and integrative medicine in lung cancer [9]. For an adequate treatment decision health care professionals should use evidence-based clinical practice guideline to support their patients for treatment options. Interestingly, a recently published study found an association to higher risk of death for patients who received complementary medicine and refuse additional conventional cancer treatment [10]. It is important to know that a therapy that used complementary medicine cannot replace a conventional therapy.

### OTCs and integrative medicines

OTC products encompass drugs, bandage and wound dressing-materials, therapeutic appliances and other materials customary in pharmacies. These four product groups have in common that they do not require a prescription from a physician and they are available in pharmacies, drug stores, internet shops, beauty shops, and sometimes in grocery stores. Not all of these products are relevant for integrative medicine (IM). IM can be defined as a holistic approach to patient care with focus on complementary health practices [11]. This paper is interested in natural or chemical substances among the OTCs, which have the potential to modify medical treatments of diseases. A recent review headed in this direction and indicated that non-prescription products used for the treatment of common conditions or symptom management influenced the health of patients in a positive way [12]. Otherwise, a recently published review shows that an irrational use may lead to consequences such as antimicrobial resistance and drug interactions [13]. However, different challenges are associated with the widespread use of OTC drugs such as adverse drug reactions or missing information about the adequate usage [5, 14].

### The OTC measurement challenge

The generally accepted framework of the Anatomical Therapeutical and Chemical (ATC) classification elaborated by the World Health Organization (WHO) does not fit to all kind of (OTC-) drugs and assistive technologies. This is particularly true for OTC-products, which do not have an ATC code of the main assortment or which ATC code does not cover all kinds of product facets (e.g. tablets, formulations, sals, composition etc.). Hence, it is virtually impossible to capture all kind of OTC-products from the main assortment by simply dropping the from-behind-counter drugs respective prescription-based drugs (Rx). Thus, the ATC classification referring to the main assortment is incomplete to reflect the total OTC drug usage. Only small changes or extensions for the main assortment would not help to overcome this gap of methodological conceptualization. However, the majority of (market) studies do not provide methodological insights into the OTC measurement concepts and probably refer to the main assortment only by neglecting the peripheral assortment [2, 4, 15].

### Aim of study

Therefore, the aim of the current study was to develop an extended measurement concept for natural and chemical substances among the OTCs. The relevance of the peripheral assortment should be explore in a stepwise manner in order to deploy the full information provided by the ATC framework. This way, we intend to describe the usage of OTC substances by private insured patients in Germany with the specific focus on integrative medicine.

## Methods

We used a cross-sectional design and analyzed claims data from PHI to overcome challenges regarding selection bias and non-representativeness [16].

### Private health insurance data

Claims data from the PHI refers to the ambulatory setting from the year 2016 [15]. The focus is on the prescriptions and drug-related receipts submitted by privately insured persons to 18 private health insurers (PHIs). The PHIs scan the prescriptions/receipts for a paperless flow of documents and save the data for further refund purposes. Once a year, the private health insurers report this consumption-related data to the central clearing-institution (Scientific Institute of Private Health Insurance, WIP) [15].

In addition, the official statistical report 2017 and the associated statistical portal delivered aggregated claims amount per risk group to enable a bound extrapolation of the sample results as a result of the bottom-up approach (details below) [17, 18].

### Scope of OTCs

The ATC classification defines the commodity groups and discriminates between the main (A) and the peripheral assortment (B). The main part A reflects the ATC-logic with the methodological flaws described earlier. This common approach only referred to the main assortment (A) and neglected the peripheral assortment (B). Therefore, both main and peripheral assortment called as commodity approach were considered for the analyses of OTCs. The peripheral assortment B encompasses a variety of commodity groups (in total 115) which are not fully relevant for an OTC description. The study group explored and assessed the relevance of each category independently according to the above-mentioned definitions of OTCs and integrative medicines. The deviating results were discussed and a pharmacist gave ultimate advice. As the outcome, a consensus list of relevant OTC-products respective commodity groups is proposed (see Additional file 1: Consensus list of relevant OTC products respective commodity groups). Finally, real claims data of the PHI from the year 2016 served as the empirical base of the proposed taxonomy.

### External data sources

The consumption-related drug data was enriched with information (variables) from ABDATA via the pharmaceutical central number (PZN) number. ABDATA collects all available pharmaceutical and economic-pharmaceutical data on drugs in Germany and makes them available to doctors, pharmacies and other institutions in a structured and permanently updated database [19].

**Fig. 1:**
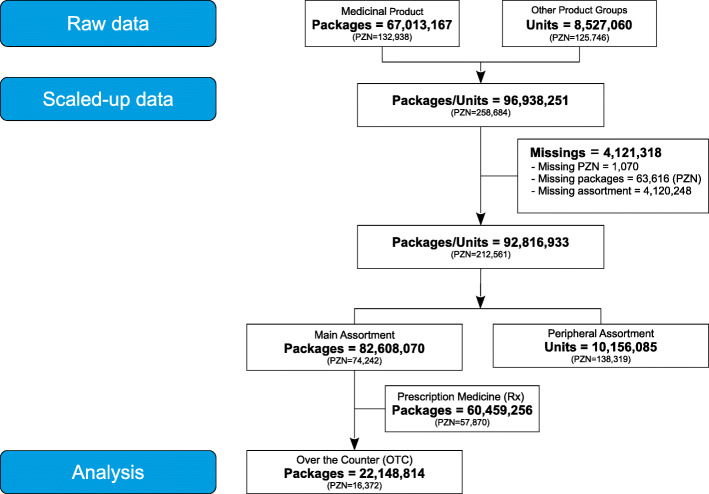
Data selection

The raw data based on 67.0 Mio. packages from medicinal products and 8.5 Mio. units from other product groups. Other product groups included bandage and wound dressing-materials, therapeutic appliances and other materials customary in pharmacies. The consolidated and scaled up data set accounted for 96.9 Mio. packages respective units with 258,684 distinct PZNs. The number of packages diminished due to missing data (4.1 Mio. packages) The remaining 92.8 Mio. packages split into the main assortment with 82.6 Mio. packages and its OTC products (22.1 Mio. packages) and the peripheral assortment with 10.1 Mio. products (see Fig. 1) to be examined.

### Extrapolation procedures

The data from 18 PHIs are claims data and do not cover all OTC-related expenses from the whole branch (non-comprehensive survey). However, in order to be able to make assumptions for the entirety of the PHIs, a scaling-up procedure should bond the empirical results of the 18 PHIs to the expenses of the 44 PHIs. The age- and gender-specific average claims amount per risks of outpatient drug expenditure were used as weights to scale up the relative distributions of the item amounts. The extrapolated number of drug packages was a result of the average packing price and the extrapolated item amount. In this way, the relationships in the age and gender structure of the entire private health insurance system was transferred to the extrapolated and bonded sample results.

### Variables

Each PZN had information about the “item amount” and the “number” of packages. The item amount reflects the pharmacy delivery price (gross), which is set by the pharmacy itself for non-prescription drugs (§ 44–46 of the German Pharmaceutical Act [20]). This price information steam from the ABDATA. Additionally, the ABDATA contributed with other variables associated with the characteristics of each OTC if available. Information of the commodity group (main and peripheral assortment), the product group, the active substance, the short name respectively the product name and the general price at the selling point in German pharmacies were used to describe the OTCs.

### Statistical analyses

A descriptive analysis assessed the OTC frequencies and sum scores of the product groups within the main and peripheral assortment. The latter refers to all commodity groups to explore the relevance of the peripheral assortment. The product level showed a selection (TOP10) of the highest selling products. Missing values were marked.

### Data protection

Analysis of claims data is consumption-oriented and does not display any individual-related information. Therefore, article 2 of the General Data Protection Regulation (EU-DSGVO) is not concerned [21]. All applicable data protection regulations were met.

## Results

The proposed commodity approach accounts for 22.1 Mio. packages among the main assortment and for 10.1 Mio. units among the peripheral assortment. In total, 32.6 Mio. units (33% from totals) were captured. The peripheral assortment of 10.1 Mio. packages should be screened for natural and chemical substances with the ability to modify medical treatment effects.

### Description of the main assortment

The analyses included 22.8 Mio. packages of OTC products among the main assortment with a market value of 361.2 Mio. €. Among these, 81.5% of these OTCs products are drugs (283.5 Mio. €). The ATC groups with the highest monetary relevance steam from diagnostics, which accounted for 45.5 € of these products (1.7 Mio. packages), cough and cold preparations caused 29.9 Mio. € (2.5 Mio. packages), psychoanaleptics achieved an amount of 24.4 Mio. € (0.3 Mio. packages) followed by ophthalmologicals with 18.0 Mio. € (1.5 Mio. packages) to cover the most prevalent medical needs of patients. All other OTC products accounted for less than 5% of the packages. However, the billing amounts of the top 10 product groups is higher than 2 Mio. € in 2016 (see Table 1).

**Table 1:** OTC consumption of the main assortment for privately insured people in 2016

Main assortment		Packages		Billing amount^**a,b**^	
3-digit code	Description	in Million	in %	in Million €	in %
V04	Diagnostic agents	1.7	7.7	45.5	12.6
R05	Cough and cold preparations	2.5	11.1	29.9	8.3
N06	Psychoanaleptics	0.3	1.4	24.4	6.7
S01	Ophthalmologicals	1.5	6.6	18.0	5.0
A06	Drugs for constipation	0.9	3.9	16.0	4.4
A12	Mineral Supplements	0.7	3.3	14.5	4.0
A07	Antidiarrheals^c^	0.7	3.2	14.3	4.0
A09	Digestives, incl. Enzymes	0.2	0.8	12.1	3.3
D01	Antifungals^d^	0.6	2.7	11.6	3.2
B03	Antianemic preparations	0.5	2.4	10.1	2.8

^a^ The billing amounts are not identical with the benefit amount (the cost reimbursement principle within the private health insurance), ^b^ The billing amount included aid and self-participation ^c^ and intestinal antiinflammatory/antiinfective agents, ^d^ for dermatological use

### Description of the peripheral assortment

In the year 2016 the privately insured demanded a total of 10.1 million products/packages of which 32.0% accounted for OTC drugs, 17.7% for bandages and wound dressing-materials, 28.7% for therapeutic appliances and 21.7% for other materials customary in pharmacies. The peripheral assortment perspective by means of the two-digit code identifies the subcategories “medical treatment needs” (BA), “medicinal products for special therapy options” (BX), “pharmaceutical food products” (BB) and “hygiene and body care products” (BC) as the most important subcategories with revenues greater than 5.6 Mio. € (see Table 2).

**Table 2:** OTC consumption of the peripheral assortment for privately insured people in 2016

Peripheral assortment		Packages		Billing amount^**a,b**^	
2-digit code	Description	in Thousand	in %	in Million €	in %
BA	Medical treatment needs	4709.2	46.4	86.1	47.3
BX	Medicinal products for special therapy options	3236.8	31.9	50.0	27.4
BB	Pharmaceutical food products	1609.6	15.8	39.5	21.7
BC	Hygiene and body care products	526.6	5.2	5.6	3.1
BF	Pharmacy supplies	34.3	0.3	0.6	0.3
BV	Medical devices pharmaceutical characters	34.3	0.3	0.2	0.1
BZ	Veterinary medicinal product	2.5	0.0	0.0	0.0
BE	Pesticides	1.3	0.0	0.0	0.0
BD	Veterinary supplies	0.9	0.0	0.0	0.0
BG	Family planning	0.6	0.0	0.0	0.0

^a^ The billing amounts are not identical with the benefit amount (the cost reimbursement principle within the private health insurance), ^b^ The billing amount included aid and self-participation

The peripheral assortment differentiated between products for humans and animals. All commodity groups referring to animals (BE, BD, BZ) can be dismissed from further conceptualization. All products referring to family planning deal with healthy people and can be excluded from further analysis. The subcategory “pharmacy supplies” (BF) refers to the process of drug preparations drugs, chemicals, containers, detergents/cleaning agents etc. These substances are irrelevant for patients. Finally, the commodity group “Medical devices of pharmaceutical characteristics” (BV) are excluded due to the proposed OTCs definition of natural and chemical substances.

The remaining subcategories “Medical treatment needs” (BA), “Medicinal products for special therapy options” (BX), “Pharmaceutical food products” (BB), and “Hygiene and body care products” (BC) are worthy to be examined in more depth. Table 3 shows the largest expenditure blocks.

**Table 3:** TOP 10 of possibly relevant OTC commodity groups for privately insured people in 2016

Peripheral assortment	5-digit code	Description	Packages		Billing amount^**a,b**^	
			in Thousand	in % from all packages	in Million €	in % from all billing amounts
Medical treatment needs (BA)	BA04D	Parenteral application	1451.7	14.4	18.0	9.9
	BA01F	Other badanging materials	365.4	3.6	17.2	9.5
	BA02A	Collection devices, catheters	499.4	5.0	7.8	4.3
	BA01A	Compresses	294.2	2.9	7.6	4.2
	BA01D	Pflasters	544.7	5.4	5.6	3.1
	BA01C	Bandages	366.4	3.6	4.5	2.5
	BA04H	Medical supplies, devices	256.8	2.5	3.8	2.1
	BA02C	Diapers, diaper pants	170.8	1.7	3.5	1.9
	BA03A	Pouches and supplies	59.2	0.6	3.1	1.7
	BA02B	Draw sheets, underlays, pads	221.2	2.2	3.0	1.7
Pharmaceutical food products (BB)	BB03Z	Miscellaneous	753.1	7.5	19.0	10.5
	BB01C	Astronaut food	210.3	2.1	9.8	5.4
	BB03D	Vitamins, minerals, combinations with other substances^c^	205.1	2.0	3.8	2.1
	BB03C	Mineral supplements	179.0	1.8	2.7	1.5
	BB03F	Gastro-intestinal treatments, digestion	46.1	0.5	1.3	0.7
	BB03A	Vitamins, monopreparations	82.9	0.8	1.2	0.6
	BB03B	Vitamins, combination preparations	42.0	0.4	0.7	0.4
	BB03G	Other phytotherapeutics, immunomodulators	14.9	0.1	0.2	0.1
	BB06B	Cough drops	27.4	0.3	0.2	0.1
	BB01E	Sports nutrition	4.2	0.0	0.1	0.1
Hygiene and body care products (BC)	BC01D	Skin- and body care	283.0	2.8	3.3	1.8
	BC01F	Hair care	37.6	0.4	0.4	0.2
	BC01C	Skin cleansing	43.4	0.4	0.4	0.2
	BC04	Sunscreen products	13.9	0.1	0.2	0.1
	BC02B	Toothpastes, mouthwashes, rising solutions	25.1	0.2	0.2	0.1
	BC01M	Toiletries, sanitary products	20.0	0.2	0.2	0.1
	BC01E	Hand- and nail care	18.5	0.2	0.1	0.1
	BC01H	Foot care	17.5	0.2	0.1	0.1
	BC02Z	Miscellaneous	17.6	0.2	0.1	0.1
	BC01N	Essential oils, tinctures, rubbing alcohol	6.4	0.1	0.1	0.0
	BC01K	Bath additives	6.5	0.1	0.1	0.0
Medicinal products for special therapy options (BX)	BX01	Homeopathy and biochemistry	2551.0	25.3	37.2	20.5
	BX02	Anthroposophical products	685.8	6.8	12.7	7.0
	BX99	Miscellaneous	0.0	0.0	0.0	0.0

^a^ The billing amounts are not identical with the benefit amount (the cost reimbursement principle within the private health insurance), ^b^ The billing amount included aid and self-participation. ^c^ Combination with other substances possible

Table 3 shows the ten products with the highest revenues for each subcategory. At the top of the group “medical treatment needs” (BA) are applications that place drugs “past the gut” (parenteral applications) with an amount of 18.0 Mio. €. However, these products and all the others from this subcategory have in common that they refer to the devices or auxiliary materials themselves. This commodity group is different from the one, which is called “Pharmaceutical food products” (BB). BB-products are used to refill e.g. parental applications and definitely meet the defined inclusion criteria.

This remains true particular for very heterogeneous items such as “Miscellaneous” on top of the list of the BB-products, because they contain natural or chemical substances. With the following items such as astronaut food, vitamins and/or minerals in various variations and combinations it becomes clear that this commodity group meets the characteristics of the ruling OTC definition of this conceptualization.

The commodity group of “Hygiene and body care” (BC) show different items essential for the daily living. However, they do not have the ability to modify treatment effects except “Essential oils, tinctures, rubbing alcohol” (BC01N) and “Bath additives” (BC01K), which are based on herbs and sals.

### Description of homeopathy/biochemistry

To learn more about the “medicinal products for special therapy options” (BX), Table 4 shows the 10 most important products of the commodity group homeopathy and biochemistry (BX01). The respective percentages referred to the aggregated quantities or item amounts within subcategory BX01, which the privately insured requested in 2016. The products referred to general injuries and wounds, sleep disorders/insomnia, respiratory tract infections and common cold. The TOP 10 of these products accounted for 24% of the billing amounts within the subcategory BX01.

**Table 4:** TOP 10 of OTC homeopathy / biochemistry for privately insured people in 2016

Product name	Field of application	Packages		Billing amount^**a,b**^	
		in Thousand	in % of homeopathy / biochemistry	in Million €	in % of homeopathy / biochemistry
TRAUMEEL	Verstauchungen, Verrenkungen, Prellungen, Blutergüsse	133.4	5.2	2.1	5.6
NEUREXAN	Nervous disorders, sleep diorders / insomnia, stress symptoms	60.3	2.4	1.3	3.6
MEDITONSIN	Common cold	59.9	2.3	0.9	2.3
VERTIGOHEEL	Dizziness, vertigo attacks	43.6	1.7	0.8	2.2
OTOVOWEN	Earache	55.5	2.2	0.8	2.1
CONTRAMUTAN	Common cold	54.2	2.1	0.7	1.9
LYMPHOMYOSOT	Edema, lymph gland neoplasia during tonsillitis	34.0	1.3	0.7	1.9
TONSIPRET	Sore throat	55.4	2.2	0.6	1.6
CALMVALERA	Nervous disorders, sleep diorders / insomnia	23.6	0.9	0.6	1.5
LYMPHDIARAL	Supportive treatment of respiratory tract infections	30.5	1.2	0.5	1.5

^a^ The billing amounts are not identical with the benefit amount (the cost reimbursement principle within the private health insurance), ^b^ The billing amount included aid and self-participation

### Description of anthroposophy

Table 5 shows the most important anthroposophical products (subcategory BX02). All anthroposophical products shared an unspecific effect on the organism. Therefore, the presented areas of application reflected a few main indications derived from the patient information leaflets. A generally high demand for mistletoe therapies, as an addition to tumor therapy was noticeable. The anthroposophical products amounted about 43% of all billing amounts in this subcategory.

**Table 5:** TOP 10 of OTC anthroposophy for privately insured people in 2016

Product name	Field of application	Packages		Billing amount^**a,b**^	
		in Thousand	in % of antrophosophy	in Million €	in % of anthroposophy
ISCADOR	Mistletoe therapies as an addition to tumor therapy	15.3	2.2	1.4	11.3
HELIXOR	Mistletoe therapies as an addition to tumor therapy	10.6	1.5	1.1	8.8
EUPHRASIA	Conjunctivities, miscellaneous	73.2	10.7	0.9	6.7
ABNOBAVISCUM	Mistletoe therapies as an addition to tumor therapy	6.5	0.9	0.6	5.0
NEURODORON	Nervous exhaustion and metabolic weakness	14.3	2.1	0.3	2.3
ISCUCIN	Mistletoe therapies as an addition to tumor therapy	4.8	0.7	0.3	2.1
AURUM	Neurasthenia, vegetative dystonia, depressive state, lack of concentration, weakness of memory	16.2	2.4	0.3	2.0
ACONIT	Joint or muscle problem, earache	21.6	3.2	0.2	1.9
HEPATODORON	Stimulation of liver or bowel action	8.8	1.3	0.2	1.6
BRYOPHYLLUM	Insomnia, irritable bladder	8.7	1.3	0.2	1.5

^a^ The billing amounts are not identical with the benefit amount (the cost reimbursement principle within the private health insurance), ^b^ The billing amount included aid and self-participation

## Discussion

Overall, the methodological approach included 22.1 Mio. packages from the main assortment and examined 10.1 Mio. packages/units from the peripheral assortment. The latter was examined thoroughly and the commodity groups “Pharmaceutical food products” (BB), “Medicinal products for special therapy options” (BX) and particular “Hygiene and body care products” (BC01N, BC01K) meet the defined requirements for OTCs relevant for integrative medicines. From the 10.1 Mio. packages, 4.8 Mio. packages/units were left. These products account for 18% taken the packages from main assortment together. Thus, generally 18% of OTC consumption is neglected by the “pure” ATC-approach and cause underestimations of the real consumption of natural and chemical substances of OTCs.

A high proportion of OTC products from the peripheral assortment was associated with the categories “medicinal products for special therapy options”. Homeopathy and anthroposophy present two special therapy options, which the private insured frequently requested in 2016. The detailed results showed that major needs (packages) and high expenditures for the application sprains, nervous restlessness, insomnia and common cold. Additionally, high expenditures caused conjunctivitis applications and mistletoe therapies within the group of anthroposophy.

### Cost, evidence and preferences

The presented costs of OTC drugs reached a considerable level for the private insurances. However, the monetary value of these OTC products do not reflect reimbursement amounts by private payers (financial support financed by the German federal government, a regional state or a municipal authority). Billing amounts are only payed if the physician prescribed OTCs and the corresponding contract/tariff covers OTC. Otherwise, the private insured become out-of-pocket payers like persons from the statutory health insurance. Regardless of the insurance type, Germans keep on buying OTCs and particularly homeopathy and anthroposophical products [2].

The medical evidence retrieved from randomized controlled trails (RCT) to show the efficacy of various homeopathy products remains unclear. Different meta-analyses showed no convincing evidence from conducted studies so far [22, 23]. The situation for anthroposophy products is similar due to a Cochrane review that showed no evidence to the application of mistletoe extracts as additional treatment in cancer therapy for overall survival or disease free survival. However, one report identified small benefits in terms of quality of life associated with a palliative cancer therapy [24, 25]. Moreover, it was found that the application of mistletoe extracts had few side effects [24, 25]. A systematic review concluded that the adverse effects of homeopathy treatment are similar to conventional medicines [26]. Furthermore, the evidence for other applications are also unclear. For the treatment of respiratory tract infections a systematic review supports the effective use of integrative medicine, especially Chinese herbal medicines [27]. For the treatment of insomnia, which is also a high prevalent condition, integrative medicine is recommended as part of cognitive-behavioral therapies [28].

The current development of studies and reviews indicate that most OTC substances are considered as something “additional” or “extra” to conventional therapies, which should be integrated carefully into common treatment regimes. However, the contrary is the case. Patients get uncontrolled access to OTC drugs regardless of the insurance type and refunding practices of insurances. Patients follow their health beliefs and hopes to define their best treatment conditions by means of additional OTCs particularly in life threating situations – and without informing the leading physician of the main therapy [29]. Thus, it seems that patients do not care about costs or evidence. They express their own preference by – at least in Germany – acting alone without the professional advice of physicians and pharmacists. A lack of medication information is the reason.

### The lack of information

As professionals do not have an overview of medications provided by only one medication plan (not two nor three), possible interactions between substances remain undetected. But very little is known about the toxic interactions between pharmaceutical treatments (e.g. cytostatics, immunospressives, etc.), OTCs and daily nutrition’s such as fruit (e.g. grapefruit) and vegetables may develop. The case of the undermining effect of grapefruit towards immunosuppressive substances has been largely described and is only one example from the field [30]. It should be possible to distinguish “harmful”, “non-harmful” and “neutral” effects from OTCs towards evidence based treatment regimes (e.g. cancer care) at least. Therefore, more research is needed to the use of OTC [31, 32]. Individuals play an active role for their own health. A crucial aspect for individuals is the level of health literacy, which included the cognitive and social skills by each individual [33]. Moreover, it has been found that the use of complementary medicine is not associated with health literacy but individuals with a lower level of health literacy prefer support by a practitioner qualified in complementary medicine [34]. It can be assumed that the adequate use of OTC-products depending on the level of health literacy.

The data of the PHI refers to nearly 90% of 8.8 million privately insured in Germany. A high external validity is very likely. All submitted prescriptions were scaled up to the average pharmaceutical expenses of 2016. A compensation of not submitted prescriptions or invoices is possible. Finally, the PHI data itself is a strength. If physicians prescribe OTCs, they belong to the general standard insurance packages of the PHI. Further analysis and reporting on OTCs alone and/or in conjunction with other substances (e.g. cancer care) is possible. Thus, further research with the developed methodological approach is possible.

### Limitations

However, typical weaknesses of claims data such as data quality issues, missing indications (e.g. diagnosis), medical outcomes, patient reported outcomes or a distinction between acute or long-term medications are present in this study. PHI related special issues deal with the refunding system. This study reported on item amounts and referred to the pharmacy-selling price. This view emphasizes the economic importance of the OTCs for the gross domestic product of one national economy. However, the revenues do not reflect the real refunding amounts of the PHIs because complete refunding of invoices depend on contract, tariff, and deductible of each individual. Additionally, it is very likely that privately insured do not submit invoices of 1 year to their PHI. Instead, they collect all invoices (not only for pharmaceuticals) related with the ambulatory setting and start calculating until the amount of invoices is higher than the expected refunding (typically a monthly premium). Additionally, the proportion of never submitted invoices due to small and irrelevant amounts remain currently unknown and are only estimable with other data sources.

## Conclusions

The analysis of OTC drugs is feasible when the OTCs of the main and the peripheral assortment is available. Reports, which only refer to the main assortment, underestimate consumption of OTC drugs. It is recommended to use the proposed commodity approach to integrate about 18% of all OTCs, which are neglected by the common ATC-based approach focusing on the main assortment only. This approach may help to identify potential users of OTCs or people in need of OTC use. In case of the highly disputed homeopathy and anthroposophy products, more research among interactions with prescriptions drugs (Rx), nutrition’s and other potentially harmful exposures is recommended. The effectiveness of the basic treatment and the non-disturbing character of the OTC should be on top of the list and clinical categories such as “harmful, non-harmful, neutral” until “additional protective (mediative)” would be very helpful for all non-professionals. The patient is highly dependent here on professional advice from physicians and pharmacist.

## Supplementary information

**Additional file 1.** Consensus list of relevant OTC products respective commodity groups.

## Data Availability

The datasets used and/or analyzed during the current study are available from the corresponding author on reasonable request.

## Abbreviations

AESGP: Association of the European Self-Medication Industry
ATC: Anatomical Therapeutic Chemical
AWMF: Association of the Scientific Medical Societies
EU-DSGVO: General Data Protection Regulation
IM: Integrative medicine
OTC: Over-the-counter
PHI: Private health insurers
PZN: Pharmaceutical central number
RCT: Randomized controlled trails
WIP: Scientific Institute of Private Health Insurance
WHO: World Health Organization
