# Supplementary material for: The usage of over-the-counter products by private insured patients in Germany – a claims data analysis with focus on complementary medicine
Source: BMC Health Serv Res. 2020 Jul 13;20:651. doi: 10.1186/s12913-020-05501-1 (PMC7359243; doi:10.1186/s12913-020-05501-1)
Supplement: Supplementary file 1 — Additional file 1. Consensus list of relevant OTC products respective commodity groups. [file 12913_2020_5501_MOESM1_ESM.docx]

**Additional file 1: Consensus list of relevant OTC products respective commodity groups**

| **Commodity group** | **Description** | **Inclusion** |
| --- | --- | --- |
| B | Peripheral Assortment | - |
| BA | Medical treatment needs | - |
| BA01 | Bandaging materials | - |
| BA01A | Compresses | - |
| BA01B | Mull, swabs | - |
| BA01C | Bandages | - |
| BA01D | Plasters | - |
| BA01E | Cotton | - |
| BA01F | Other bandaging materials | - |
| BA02 | Incontinence | - |
| BA02A | Collection devices, catheters | - |
| BA02B | Draw sheets, underlays, pads | - |
| BA02C | Diapers, diaper pants | - |
| BA03 | Ostomy products | - |
| BA03A | Pouches and supplies | - |
| BA03B | Irrigators and supplies | - |
| BA03C | Skin protection products | - |
| BA04 | Medical-technical commodities | - |
| BA04A | Protection and support of body parts | - |
| BA04B | Compression stockings | - |
| BA04C | Decubitus prophylaxis/prevention | - |
| BA04D | Parenteral Application | - |
| BA04E | Measuring devices, thermometers | - |
| BA04F | Inhalers | - |
| BA04G | Application of heat and cold | - |
| BA04H | Medical supplies, devices | - |
| BA04K | Diagnostic tools and supplies | - |
| BA04L | Information carriers | - |
| BA04M | Daily living aids/auxiliaries for physical disabilities | - |
| BA04N | Suppport stockings | - |
| BA04Z | Miscellaneous | - |
| BB | Pharmaceutical food products | X |
| BB01 | Dietetics, diet products | X |
| BB01A | Reduction diets | X |
| BB01B | Diabetic food | X |
| BB01C | Astronaut food | X |
| BB01D | Sweeteners, sugar substitutes | X |
| BB01E | Sports nutrition | X |
| BB01Z | Miscellaneous | X |
| BB02 | Infant food | X |
| BB02A | Convenience complete food | X |
| BB02B | Healing food, therapeutic food, curative nutrition | X |
| BB02C | Preserved food, baby food (jars) | X |
| BB02D | Infant teas, juices | X |
| BB02Z | Miscellaneous | X |
| BB03 | Dietary supplements | X |
| BB03A | Vitamins, monopreparations | X |
| BB03B | Vitamins, combination preparations | X |
| BB03C | Mineral supplements | X |
| BB03D | Vitamins, minerals, combinations with other substances | X |
| BB03E | Nutritional supplements, geriatrics | X |
| BB03F | Gastro-intestinal treatments, digestion | X |
| BB03G | other phytotherapeutics, immunomodulators | X |
| BB03Z | Miscellaneous | X |
| BB04 | Standard apothecary drinks | X |
| BB04A | Healing water | X |
| BB04B | Juices | X |
| BB04C | Medicinal wines | X |
| BB05 | Ready-to-serve tea | X |
| BB05A | Home made tea | X |
| BB05B | Medicinal teas | X |
| BB06 | Confectionery | X |
| BB06A | Candies, glucose | X |
| BB06B | Cough drops | X |
| BB06C | Fruit bars, bars | X |
| BC | Hygiene and personal care / Hygiene and body care products | - |
| BC01 | Medicinal body care products | - |
| BC01A | Decorative cosmetics | - |
| BC01B | Men's series, shaving | - |
| BC01C | Skin cleansing | - |
| BC01D | Skin- and body care | - |
| BC01E | Hand- and nail care | - |
| BC01F | Hair care | - |
| BC01G | Lip care | - |
| BC01H | Foot care | - |
| BC01K | Bath additives | X |
| BC01L | Antiperspirants | - |
| BC01M | Toiletries, sanitary products | - |
| BC01N | Essential oils, tinctures, rubbing alcohol | X |
| BC01O | Depilatories, bleaching agents | - |
| BC01Z | Miscellaneous | - |
| BC02 | Dental hygiene and oral care | - |
| BC02A | Toothbrushes, oral irrigators, dental floss, dental sticks | - |
| BC02B | Toothpastes, mouthwashes, rinsing solutions | - |
| BC02C | Care of 3. teeth | - |
| BC02Z | Miscellaneous | - |
| BC03 | Care products for babies and children | - |
| BC03A | Diapers | - |
| BC03B | Skin care | - |
| BC03C | Soothers, suckers, bottles | - |
| BC03Z | Miscellaneous | - |
| BC04 | Sunscreen products | - |
| BC05 | Contact lens products | - |
| BC06 | Repellents | - |
| BD | Veterinary supplies | - |
| BE | Pesticides | - |
| BE01 | Plant protection products | - |
| BE02 | Disinfectants/disinfection agents | - |
| BE03 | Toxins and lures/attractants | - |
| BF | Pharmacy supplies, formulation, chemicals | - |
| BF01 | Drugs, chemicals | - |
| BF02 | Containers | - |
| BF03 | Detergents/cleaning agents | - |
| BF99 | Miscellaneous | - |
| BG | Family planning | - |
| BG01 | Condoms, diaphragms | - |
| BG02 | (Vaginal) pessaries, cremes | - |
| BG03 | Pregnancy tests | - |
| BG04 | Ovulation tests | - |
| BV | Medical devices with pharmaceutical characters | - |
| BX | Medicinal products for special therapy options | X |
| BX01 | Homeopathy and biochemistry | X |
| BX02 | Anthroposophical products | X |
| BX99 | Miscellaneous | X |
| BZ | Veterinary medicinal products | - |
